# Altered fractalkine cleavage potentially promotes local inflammation in NOD salivary gland

**DOI:** 10.1186/ar2441

**Published:** 2008-06-19

**Authors:** Manon E Wildenberg, Cornelia G van Helden-Meeuwsen, Hemmo A Drexhage, Marjan A Versnel

**Affiliations:** 1 Department of Immunology, Erasmus MC, P.O. Box 2040, 3000 CA Rotterdam, The Netherlands

## Abstract

**Introduction:**

In the nonobese diabetic (NOD) mouse model of Sjögren's syndrome, lymphocytic infiltration is preceded by an accumulation of dendritic cells in the submandibular glands (SMGs). NOD mice also exhibit an increased frequency of mature, fractalkine receptor (CX3C chemokine receptor [CX3CR]1) expressing monocytes, which are considered to be precursors for tissue dendritic cells. To unravel further the role played by fractalkine-CX3CR1 interactions in the salivary gland inflammation, we studied the expression of fractalkine in NOD SMGs.

**Methods:**

We studied protein expression using Western blot analysis of whole tissue lysates. Protease activity was measured in salivary gland tissue lysates using fluorimetric substrates. Digestive capacity of enzymes was determined by *in vitro *incubation of recombinant enzyme and fractalkine, followed by protein staining and Western blot.

**Results:**

Fractalkine was detected in salivary glands of both NOD and control mice at all ages. Western blot analysis showed fractalkine cleavage with increasing age, which was more pronounced in NOD mice. This cleavage resulted in a decrease in the 31 kDa form of the protein, and the generation of an approximately 19 kDa band. Furthermore, in NOD animals older than 15 weeks, we noted the presence of a unique approximately 17 kDa fragment. This cleavage was organ specific, because it did not occur in brain or pancreas. Increased gelatinase and α-secretase activity were detected in NOD SMG and contributed to cleavage of the 31 kDa protein. Because aberrant cleavage products may induce autoimmunity, we studied the presence of autoantibodies against fractalkine. Indeed, NOD mice exhibited significantly more antibodies against fractalkine than did control animals.

**Conclusion:**

These data indicate that aberrant proteolytic activity in the NOD SMG results in increased fractalkine cleavage and generation of a unique fractalkine fragment. This specific cleavage may contribute to autoimmunity.

## Introduction

The nonobese diabetic (NOD) mouse is a frequently used spontaneous animal model for the development of Sjögren's syndrome (SjS). Similar to SjS patients, these mice develop lymphocytic infiltrates in their salivary glands, which leads to gland destruction and decreased salivary flow. The development of infiltrates is preceded by an accumulation of dendritic cells (DCs). Given the key role played by DCs in the initiation of immune responses, their accumulation as one of the early events in SjS indicates that DCs are involved in the pathogenesis of the disease [1,2]. The precise cause of DC accumulation remains to be elucidated, but alterations in monocytes, which are considered to be a precursor population for DCs, have been described in the NOD mouse [3,4]. In particular, the subset of Ly-6C^low ^monocytes is significantly expanded in the NOD circulation [3]. This subset is thought to be a mature population that preferentially develops into tissue DCs [5], suggesting a link between these cells and the DC accumulation observed in the salivary gland of NOD mice.

Apart from the low expression of Ly-6C, the mature monocyte subset is further characterized by low expression of CC chemokine receptors 1 and 2, and high expression of CX3C chemokine receptor (CX3CR)1, the fractalkine receptor [6]. Fractalkine is the sole member of the CX3C chemokine family, first described in brain tissue [7,8]. It differs from other chemokines not only in its structure and relatively large size, but also in the fact that it occurs in both membrane bound and soluble forms. The membrane bound form functions as an adhesion molecule, whereas the soluble form is strongly chemotactic for monocytes and T cells [9,10].

Given the increase in mature Ly-6C^low ^monocytes in the NOD circulation, their propensity to develop into DCs and the importance of fractalkine in chemoattraction of Ly-6C^low ^monocytes, we studied the expression of fractalkine in NOD salivary glands. Although fractalkine was present in salivary glands of both NOD and control mice, a unique fragment of the protein was observed in NOD mice. Such a fragment may contribute to disruption of tolerance to fractalkine, resulting in an autoimmune response. Indeed, an anti-fractalkine antibody response in the NOD mouse is identified here.

## Materials and methods

### Animals

NOD/LTj mice were bred in our own facility. Mice were tested for diabetes twice weekly and excluded from experiments when positive. C57BL/6 and BALB/c mice were obtained from Harlan (Horst, The Netherlands). All mice were housed under specific pathogen-free conditions and were fed standard chow and water *ad libitum*. Female mice aged 5 to 25 weeks were used in all experiments. All experimental procedures were approved by the Erasmus University Animal Ethical Committee.

### Tissue lysates and protease activity

Submandibular glands (SMGs) and pancreases were removed and cleared of adipose tissue and lymph nodes. Tissue was placed in phosphate-buffered saline, or phosphate-buffered saline containing a protease inhibitor cocktail (Complete mini protease inhibitor tablets; Roche, Woerden, The Netherlands) where indicated, and homogenized by mechanical disruption followed by ultrasound sonification. Finally, lysates were cleared by centrifugation. Caspase-3 and gelatinase (matrix metalloprotease [MMP]-2/MMP-9) activity were measured using EnzCheck assay kits (Invitrogen, Eugene, OR, USA) in accordance with the manufacturer's instructions. α-Secretase activity was measured using a fluorogenic substrate (10 μg/reaction, alpha-secretase substrate II; Merck, Whitehouse Station, NJ, USA), as indicated by the manufacturer. All results are shown as arbitrary units relative to the total amount of protein, as measured by Bradford analysis (BioRad, Hercules, CA, USA).

### Western blot

Protein content of lysates was measured by Bradford analysis (BioRad) and 50 μg of each lysate was loaded onto a 15% SDS-PAGE gel and run under reducing conditions. For determination of protein size, the Kaleidoscope-prestained standard was used (BioRad). Afterward, protein was transferred to an Immobilon-P membrane (Millipore, Billerica, MA, USA). Membranes were then incubated with anti-fractalkine antibody (C-20, 1 μg/ml; Santa Cruz, Santa Cruz, CA, USA) followed by Donkey-anti-Goat-HRP (80 ng/ml; Santa Cruz). Expression was detected by enhanced chemiluminescence (ECL) analysis (Amersham, Piscataway, NJ, USA). For detection of autoantibodies, tissue lysates known to contain specific forms of fractalkine were loaded onto a 15% SDS-PAGE gel. After transfer, membranes were incubated with IgG purified from mouse serum by protein G-column separation or with total serum. This was followed by Rabbit-anti-mouse-HRP (1:20,000; Dako, Glostrup, Denmark) and ECL analysis.

### *In vitro *digestion assay

MMP-2 (65 ng/reaction; Chemicon, Temecula, CA, USA) and MMP-9 (140 ng/reaction; R&D Systems Inc., Minneapolis, MN, USA) were activated by 1 mmol/l AMPA in 10 mmol/l CaCl_2_, 100 mmol/l NaCl, and 50 mmol/l (Tris/HCl pH 7.5). Caspase-3 (10 ng/reaction; Gentaur, Brussels, Belgium) was diluted in 10 mmol/l PIPES, 2 mmol/l EDTA, 0.1% CHAPS and 5 mmol/l DTT (pH 7.4). A disintegrin and metalloprotease domain (ADAM)-10 and ADAM-17 (both 500 ng/reaction; R&D systems Inc.) were diluted in 50 mmol/l HEPES, 5 μmol/l ZnCl_2_, 0.01% Brij-0 [pH 7.5]). Enzyme activity was confirmed by fluorimetric assay as described above. Active enzyme was then incubated with brain lysate (20 μg protein/reaction) for 2 hours at 37°C. Digestion was stopped by adding reducing sample buffer and heating to 99°C. Protein analysis was carried out using Western blot as described above.

### Statistical analysis

Bars represent mean, and error bars represent standard deviation. For comparison of means Student's *t*-test was used, and for comparison of frequencies Fisher's exact test was calculated, both using SPSS software (SPSS Inc., Chicago, IL, USA).

## Results

### Fractalkine is present in NOD submandibular glands and cleaved with increasing age

**Figure 1:**
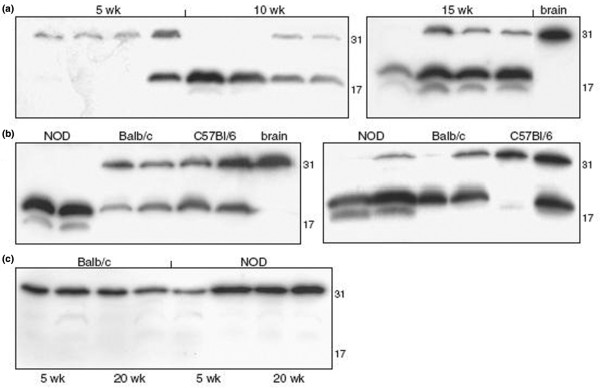
Altered fractalkine cleavage in NOD salivary gland. Whole tissue lysates were prepared in phosphate-buffered saline (PBS) supplemented with protease inhibitor by mechanical homogenization followed by sonification. Lysates were then analysed by Western blotting. A representative picture of six individual experiments is shown. **(a) **Submandibular gland (SMGs) of 5-, 10- and 15-week-old nonobese diabetic (NOD) mice. **(b) **SMGs of 10-week-old (left) and 20-week-old (right) NOD, C57BL/6 and Balb/c mice. **(c) **Pancreas of 5- and 20-week-old NOD and Balb/c mice. Numbers represent mass (kDa), as determined using a molecular weight marker.

To determine the presence of fractalkine at the protein level, expression was studied in SMG tissue lysates by Western blot. Because expression of fractalkine has been clearly described in normal brain [11,12], this was used as a reference sample. Analysis of SMG tissue from young (age 5 weeks) NOD mice revealed expression of the protein at a molecular weight similar to that observed in brain lysates (31 kDa). However, when salivary glands of 15-week-old mice were studied, additional bands were observed at about 19 kDa and about 17 kDa (Figure 1a). Also, the 31 kDa band was less intense or even undetectable at these time points, indicating cleavage of fractalkine with increasing age in the salivary glands of NOD mice.

### Fractalkine cleavage is altered in NOD compared with control strains

When control mice were studied, the profile of fractalkine cleavage differed markedly from that in NOD mice. At age 5 weeks no clear differences were found, with the expression of only the 31 kDa form of the protein in both control strains. At 10 weeks, the approximately 19 kDa band began to appear in C57BL/6 and BALB/c mice. However, whereas expression of the 31 kDa band frequently disappeared in NOD mice at this age, this band was still clearly present in the control strains (Figure 1b). The additional approximiately 17 kDa form of fractalkine, which was found in NOD mice, never appeared in either C57BL/6 or BALB/c mice at any time point up to 20 weeks (Figure 1b). These results indicate that the profile of the cleavage process differs between NOD and control strains, and that the specific cleavage in NOD SMG results in the generation of a unique fractalkine fragment in the NOD mouse.

### Cleavage of fractalkine does not occur in NOD pancreas

The NOD mouse not only develops sialoadenitis but also autoimmune insulitis. Similar to the salivary glands, lymphocytic infiltration in the pancreas is preceded by an accumulation of DCs. Because the sialoadenitis and pancreatitis observed in NOD mice are usually considered to be the result of at least partly overlapping defects, expression of fractalkine in the pancreas was studied and compared with that of the SMG. In both NOD and control mice, expression of the 31 kDa form of fractalkine was detected at all ages tested. However, smaller products were not observed at any age, either in NOD or in the control strains (Figure 1c). This indicates that the increase in protease activity is SMG specific.

### ADAM-17 induces fractalkine cleavage, but ADAM-10 and caspase-3 do not

**Figure 2:**
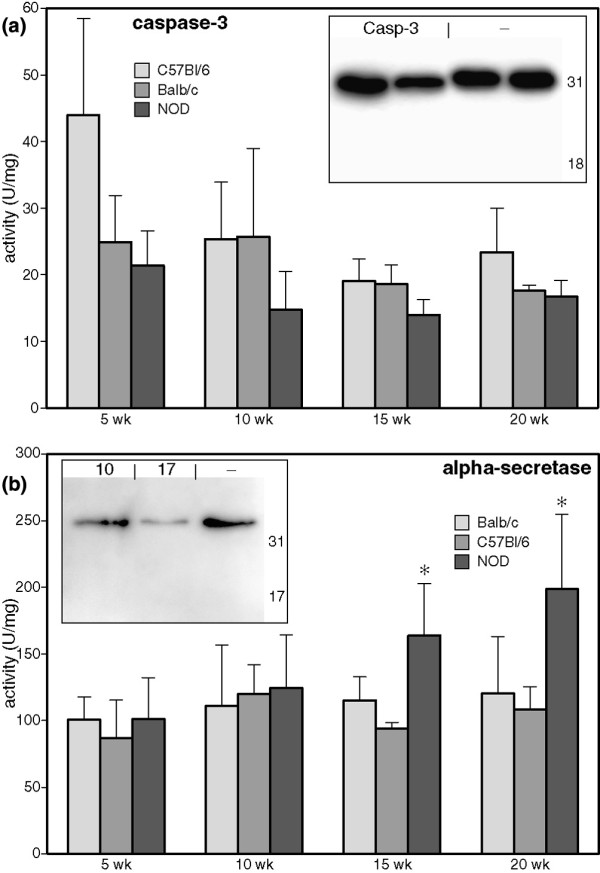
ADAM-17 but not caspase-3 cleaves 31 kDa fractalkine. **(a) **Caspase-3 and **(b) **α-secretase activities were measured in tissue lysates prepared in phosphate-buffered saline without protease inhibitors using a fluorimetric assay. Bars represent mean, error bars represent standard deviation (*n* = 5). * *P *< 0.05. Recombinant mouse caspase-3 (panel a) and a disintegrin and metalloprotease domain (ADAM)-10 or -17 (panel b) were incubated with brain lysate and analysed by Western blotting (*n* = 4). Numbers represent mass (kDa), as determined using a molecular weight marker.

Altered protein breakdown has previously been described in the SMG of NOD mice, a specific example being the autoantigen α-fodrin, and this was linked to caspase-3 activity [13-15]. Therefore, caspase-3 activity was measured in whole salivary gland lysates. Although activity could be detected at all ages, no increase in caspase-3 activity was observed in the NOD mouse (Figure 2a). Furthermore, when brain lysates containing the 31 kDa form of fractalkine were incubated with recombinant caspase-3, no digestion of fractalkine was observed (Figure 2a [insert]).

Another family of proteases involved in regulating fractalkine levels are the so-called α-secretases. These include ADAM-10 and ADAM-17, which have been shown to cleave fractalkine under steady-state and inflammatory conditions, respectively [16,17]. When measured in whole salivary gland lysates, α-secretase activity was significantly increased in NOD mice from the age of 15 weeks onward (Figure 2b). When incubated with brain lysate *in vitro*, ADAM-17 but not ADAM-10 was capable of cleaving 31 kDa fractalkine. However, the approximately 19 and 17 kDa bands did not appear (Figure 2b [insert]), and neither did any smaller fractalkine fragments down to 6 kDa (data not shown). This indicates that although ADAM-17 may contribute to the fractalkine cleavage observed in NOD SMG, other proteases probably result in the generation of the approximately 19 kDa and 17 kDa fragments.

### MMP-9 activity leads to cleavage of 31 kDa fractalkine

**Figure 3:**
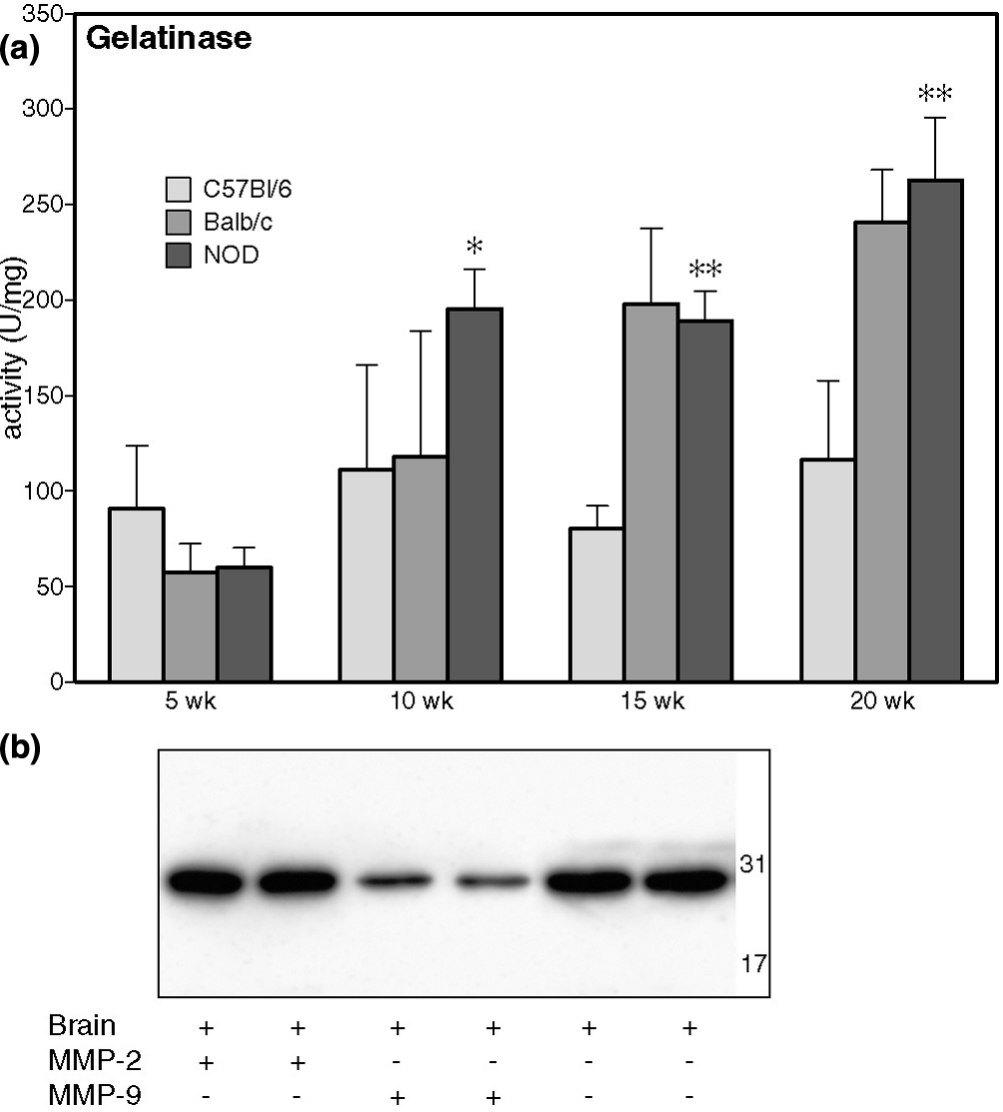
MMP-9 cleaves 31 kDa *in vitro*. **(a) **Gelatinase activity was measured in tissue lysates prepared in phosphate-buffered saline without protease inhibitors using a fluorimetric assay; bars represent mean, and error bars represent standard deviation. *P < 0.05 versus C57BL/6 and Balc/c; **P < 0.05 versus C57BL/6 (*n* = 5). **(b) **Recombinant mouse matrix metalloprotease (MMP)-2 and MMP-9 were incubated with brain lysate and analysed by Western blotting (*n* = 3). Numbers represent mass (kDa), as determined using a molecular weight marker.

Increased expression of MMP-9 has been described in the salivary glands of SjS patients, and RNA levels of both MMP-2 and MMP-9 are increased in the SMG of NOD mice [18-20]. Because fractalkine has been shown to be a ligand for MMP-2 [21], increased activity of these metalloproteases may be involved in the altered proteolysis of fractalkine. When measured in whole salivary gland lysates, gelatinase (combined MMP-2 and MMP-9) activity was increased in NOD mice aged 10 weeks – the time point at which the increased cleavage of fractalkine in the NOD first becomes apparent (Figure 3a). Furthermore, incubation of brain lysates with recombinant MMP-9, but not with MMP-2, resulted in a clear reduction in the 31 kDa form of fractalkine (Figure 3b). However, the approximately 19 and 17 kDa bands did not appear in this experiment, and neither did any smaller fragments down to 6 kDa (data not shown). This indicates that in addition to MMP-9, other proteases are likely to contribute to the fractalkine cleavage observed *in vivo*.

### Presence of autoantibodies against fractalkine in NOD

**Figure 4:**
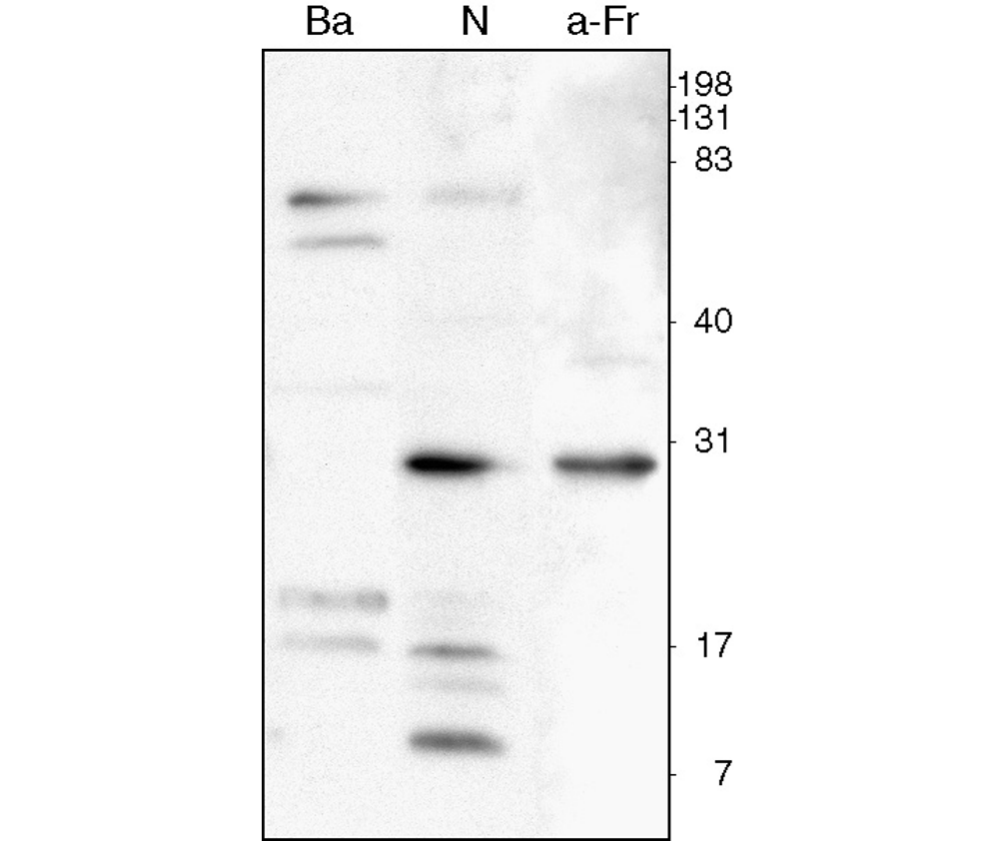
Autoantibodies against fractalkine are present in NOD mice. Tissue lysates prepared in phosphate-buffered saline supplemented with protease inhibitors were run on an SDS gel and transferred to an Immobilon membrane (Millipore, Billerica, MA, USA). Membranes were then incubated with purified IgG from either NOD (N) or Balb/c (Ba) mice. Binding of autoantibodies was detected by anti-mouse-HRP and electrochemical luminescence (ECL) analysis. Presence of specific fractalkine fragments in the lysate was detected using a polyclonal anti-fractalkine antibody (a-Fr). Numbers represent mass (kDa), as determined using a molecular weight marker.

Altered proteolysis of α-fodrin in NOD results in the generation of an autoantigen and the formation of autoantibodies [14]. Therefore, the occurrence of autoantibodies against fractalkine was studied by testing the reactivity of mouse serum with blotted brain lysate containing the 31 kDa form of fractalkine. In the serum of young (5 weeks) animals, reactivity against the 31 kDa protein could not be detected. However, in the serum of animals older than 15 weeks, antibodies against a protein running at 31 kDa were detected in 10 out of 14 NOD mice (Figure 4). In control animals, this was the case in significantly fewer (one out of six; *P *< 0.05). Similar results were obtained with purified IgG and total serum (data not shown). Fractalkine specificity of the anti-31 kDa band was confirmed by blotting against recombinant fractalkine (data not shown). These results indicate that fractalkine indeed becomes an autoantigen in the NOD mouse.

## Discussion

NOD mice exhibit an abnormal breakdown of fractalkine in salivary glands, which results in the generation of a unique fragment. This breakdown did not occur in pancreas, indicating that the phenomenon is organ specific and not a result of local inflammation. Altered proteolytic cleavage in NOD salivary glands has previously been described for α-fodrin and parotid secretory protein [13,14,22,23]. In the case of α-fodrin, proteolysis is caused by the apoptotic enzyme caspase-3 [13]. Although caspase-3 was detected in SMGs from mice of all ages, activity was not increased in NOD compared with control animals. Furthermore, caspase-3 did not cleave 31 kDa fractalkine *in vitro*. Two proteases described to be involved in the physiological shedding of fractalkine are ADAM-10 and ADAM-17 [16,17]. However, although the joint activity of these enzymes did increase in NOD mice at older ages (> 15 weeks) and ADAM-17 was capable of cleaving 31 kDa fractalkine *in vitro*, this did not results in the generation of the approximately 19 and 17 kDa bands.

Previous reports describe the abnormal breakdown of extracellular matrix components in SjS salivary glands, and this was linked to increased activity of MMPs [24]. In particular, expression of MMP-9 has consistently been found to be increased in salivary glands of SjS patients. In NOD mice, increased expression of this metalloprotease in SMG was reported in old (> 20 weeks) animals [18-20,24-27]. Our study shows that MMP-9 activity has already increased at around 10 weeks of age, similar to the time point at which cleavage of fractalkine was first observed. Additionally, MMP-9 has been shown to be capable of degrading the 31 kDa form of fractalkine. However, the characteristic approximately 17 and 19 kDa forms did not appear. When fractalkine was incubated with MMP-9 and analyzed by total protein staining, neither the approximately 19 and 17 kDa fragments nor smaller fragments were detected (data not shown). This suggests that MMP-9 cleavage results in very small fragments, at least *in vitro*. Hence, although both ADAM-17 and MMP-9 are involved in the degradation of fractalkine, other proteases are likely to be responsible for the generation of the approximately 19 and 17 kDa fragments *in vivo*. Candidates for this function include members of the caspase family, such as caspase-1 and caspase-11, both of which are increased in the salivary glands under inflammatory conditions [28,29]. Also, granzyme B may be involved, because this protease has been shown to generate antigenic fragments by cleaving α-fodrin, La protein and muscarinic receptor 3 [30,31], all of which are known autoantigens in SjS.

The precise nature of the fractalkine fragments described in this study remains to be further elucidated. Attempts to isolate the fractalkine fragments from SMG lysates were unsuccessful because of the abundant presence of other proteins in the total gland lysates. However, the antibody used to detect fractalkine in this study recognizes the carboxyl-terminal end, indicating that both the approximately 19 and 17 kDa fragments contain this part of the protein. One hypothesis is that the approximately 19 and 17 kDa fragments are the remaining (carboxyl-terminal) fragments after shedding of the amino-terminal part of the protein. This amino-terminal part contains the chemokine domain, which in this model could be released into the circulation and enhance monocyte chemoattraction to the salivary glands. Previous studies have shown that fractalkine is indeed a substrate for metalloproteinases, resulting in the generation of a highly active chemotactic fragment [32]. Interestingly, the fractalkine receptor positive population of Ly-6C^low ^monocytes is increased significantly in NOD mice [3]. This population preferentially develops in tissue DCs [33], and fractalkine receptor positive cells resembling DCs were observed in murine salivary glands (HC Reinecker, personal communication). This supports the idea of chemotactic fractalkine contributing to the accumulation of DCs in NOD salivary glands. To investigate further this contribution, it would be interesting to cross CX3CR1-deficient mice [34] back to the NOD background, and determine the effect on monocyte infiltration and DC accumulation.

In the case of α-fodrin, the altered cleavage in pSjS and NOD salivary glands results in an autoantigenic protein and the generation of autoantibodies as well as a specific T-cell response [14,35]. Importantly, when cleavage of α-fodrin is inhibited, pathology is prevented [36], indicating the significance of this process. In this study we showed that the abnormal cleavage of fractalkine is also accompanied by the occurrence of autoantibodies. In NOD mice, antibodies recognizing 31 kDa fractalkine were present from about 10 weeks and increased with age. In control mice, these autoantibodies were found in significantly fewer animals, and at lower concentrations. It is tempting to speculate that the abnormal breakdown of fractalkine in NOD SMG contributes to a break of tolerance against this self protein, thus resulting in the formation of autoantibodies. The source of fractalkine in the NOD SMG remains to be elucidated, but studies in human salivary gland show that fractalkine is expressed in glandular epithelium as well as ductal structures [37]. An anti-fractalkine response would therefore be targeted directly against the salivary gland epithelium, and contribute to destruction of the salivary gland tissue.

## Conclusion

Increased proteolytic activity in the salivary gland of NOD mice leads to the generation of aberrant fractalkine fragments. These may enhance the ongoing inflammation by functioning as autoantigens. Furthermore, the enhanced cleavage of fractalkine is hypothesized to result in increased chemotaxis. This dual role for fractalkine in local inflammation in SjS salivary glands indicates that fractalkine may be an interesting target for future therapy.

## Abbreviations

ADAM = a disintegrin and metalloprotease domain; CX3CR = CX3C chemokine receptor; DC = dendritic cell; ECL = enhanced chemiluminescence; MMP = matrix metalloprotease; NOD = nonobese diabetic; SjS = Sjögren's syndrome; SMG = submandibular gland.

## Competing interests

The authors declare that they have no competing interests.

## Authors' contributions

MEW designed the study, carried out the protease activity assays and was responsible for writing of the manuscript. CvHM carried out the Western Blot experiments and revised the Materials and methods section of the manuscript. HAD provided critical revisions to the manuscript. MAV directed the project and provided critical revisions of the manuscript. All authors read and approved the final manuscript.
